# Health utilities using SF-6D scores in Japanese patients with chronic hepatitis C treated with sofosbuvir-based regimens in clinical trials

**DOI:** 10.1186/s12955-017-0598-8

**Published:** 2017-01-31

**Authors:** Zobair Younossi, Maria Stepanova, Masao Omata, Masashi Mizokami, Mercedes Walters, Sharon Hunt

**Affiliations:** 1 Department of Medicine, Center for Liver Diseases, Inova Fairfax Hospital, Falls Church, VA USA; 2 Department of Medicine, Beatty Liver and Obesity Research Program, Inova Fairfax Hospital, Falls Church, VA USA; 3 Yamanashi Prefectural Hospital Organization, Yamanashi, Japan; 4 Kohnodai Hospital, National Center for Global Health and Medicine, Chiba, Japan; 5 Center for Outcomes Research in Liver Diseases, Washington, DC USA; 6 Betty and Guy Beatty Center for Integrated Research, Claude Moore Health Education and Research Building, 3300 Gallows Road, Falls Church, VA 22042 USA

**Keywords:** HCV, Sofosbuvir, Ribavirin, Interferon, Ledipasvir, Health-related quality of life, Health utilities

## Abstract

**Background:**

Health utilities are preference-based measures for health states which are typically used in economic analyses to estimate quality-adjusted life years. Our aim is to report the standard SF-6D health utility scores in Japanese patients with hepatitis C virus (HCV) during treatment with different regimens.

**Methods:**

Japanese patients were enrolled in clinical trials of sofosbuvir (SOF) used in combination with or without ledipasvir (LDV) and/or ribavirin (RBV). The SF-6D health utility scores were calculated at multiple time points from the SF-36 instrument.

**Results:**

Four hundred ninety-four patients with HCV (genotype 1 and 2) were enrolled: 19% with cirrhosis, 48% with a prior history of anti-HCV treatment. Of those, 153 received SOF + RBV, 170 received LDV/SOF + RBV, 171 received LDV/SOF for 12 weeks; the SVR rates were: 97, 98 and 100%, respectively. Patients treated with the three regimens had similar SF-6D scores before treatment (*p* = 0.87): 76.1 ± 11.5. During treatment with RBV containing regimen, patients experienced a decrement in their health utility scores to 74.3 ± 12.5 by the end of treatment (*p* = 0.03), while patients treated with RBV-free LDV/SOF had their SF-6D scores improved to 79.2 ± 12.8 after 12 weeks of treatment (*p* = 0.0004). At post-treatment week 12, in patients who achieved SVR-12, the SF-6D scores were similar between the treatment regimens (*p* = 0.36), and an average improvement of +1.4 points from baseline (*p* = 0.01) was noted. In multivariate analysis, the use of RBV was independently associated with lower utility score during treatment (beta = 4.7 ± 1.6, *p* < 0.0001).

**Conclusion:**

Health utilities are lower in Japanese HCV patients and tend to improve after clearance of infection.

## Background

Hepatitis C infection (HCV) causes a systemic disease with both hepatic and extrahepatic manifestations [1, 2], and also affects patient-reported outcomes (PROs) by causing significant fatigue, a decrease patients’ physical and mental functioning (measured by health-related quality of life (HRQL) survey tools) and negatively affecting worker productivity [2–6].

Introduction of the new all-oral interferon-free direct-acting antiviral regimens (DAAs) has changed the landscape of treatment and its associated outcomes. Presently, the remarkably high cure rates of these new therapies has allowed attention to shift to investigating the impact of treatment on PROs. In fact, recent studies have reported that PROs have been significantly positively affected [6–9]; in particular, in some patient groups, these positive changes occurred within 2 weeks of treatment [10].

Although interferon (IFN)-based regimens have been the historical regimens for treatment of HCV in Japan, the newer regimens free of IFN were recently approved and are now widely used in Japan [11–14]. Despite the successful outcomes of the new DAAs, the cost of treatment has caused great concern and discussion about who should receive treatment and during what stage of disease. As a result, a number of cost-effectiveness studies have been published highlighting that treatment with the DAAs is cost-effective for both the patient and the society [15–18]. However, the majority of these studies have been carried out in the Western and European countries leaving a gap of knowledge of how these results would transfer to the Asian population, especially given that HCV is the most common cause of chronic liver disease in Japan leading to high rates of cirrhosis and hepatocellular carcinoma [19, 20]. So far, to the best of our knowledge, there are no systematically collected health utility data on the impact of treatment to determine cost-effectiveness and QALYs gained as a result of using the new DAA regimens within the Japanese population. Therefore, the purpose of this study was to estimate health utility scores for the Japanese population so that cost-effectiveness studies can be carried out for this population.

## Methods

We used the exploratory endpoints data collected in two multicenter phase 3 clinical trials of sofosbuvir-based regimens for treatment of chronic hepatitis C (GS-US-337-0113 and GS-US-334-0118). These trials were conducted in Japan in 2013–2015 [12, 13]. In both trials, otherwise healthy (i.e., without current or prior history of any clinically-significant illness other than HCV) treatment-naïve or treatment-experienced HCV patients of both genders with or without compensated cirrhosis were enrolled to receive sofosbuvir (400 mg) in combination with ribavirin (weight-based 1000/1200 mg) for HCV genotype 2, or a fixed-dose combination of ledipasvir and sofosbuvir (90 mg/400 mg) with or without ribavirin for HCV genotype 1. All studied treatment regimens lasted for 12 weeks. The details of the study design for the original trials, including inclusion and exclusion criteria, and the safety and efficacy results have been published [12, 13]. For this study, similarly to our prior report for that cohort [21], using extensive medical history collected at screening for all enrolled participants, we identified patients with pre-treatment history of depression or mood disorders, anxiety or panic disorders, insomnia or sleep disorders, and type 2 diabetes or hyperglycemia, and then tested these conditions as independent predictors of the utility scores as described below.

Patients with detectable HCV RNA at post-treatment week 4 were not followed-up at subsequent follow-up visits. Patients were considered to have achieved sustained virologic response (SVR-12) if they had undetectable HCV RNA at post-treatment week 12.

### Health utility scores

In general, a health utility score is a single summary measure of patients’ preference for a certain state of health which is calculated by applying a preference-based weight to each patient’s health status; those preference-based weights are typically calculated in an a priori development or validation study for a specific health utility metric. There exist a number of instruments designed specifically for calculation of health utility scores (HUI-2, EQ-5D, etc.) Notably, since most popular HRQL instruments, including SF-36, do not include any patients’ preferences to their scoring algorithms, the HRQL scores cannot be directly used in the role of utility scores. Thus, when specific health utility instruments are not available, regression-based methods for estimating health utility scores from the HRQL scores are typically used. One of such widely used approximations of health utility scores is SF-6D metric which can be calculated from SF-36; a number of parametric and non-parametric algorithms have been published and validated [22–24].

In this study, patients completed the Short Form-36 version 2 (SF-36v2) questionnaire in their native language at baseline, during, and after treatment; we used the SF-36 data to derive participants’ SF-6D health utility scores by a Bayesian non-parametric algorithm [22]. Also, for the purpose of presentation, the SF-6D scores were transformed from a conventional 0–1 to a 0–100 scale by multiplying the scores by 100.

### Statistical analysis

The baseline health utility scores of Japanese patients were summarized and compared across the treatment regimens using the Kruskal-Wallis non-parametric test. The changes (decrements or improvements) in the utility scores from patients’ own baseline levels were calculated for each patient at each study time point, and the median changes were compared to zero (the null hypothesis of no change) by a sign rank test. Only *p*-values of 0.05 or less were considered statistically significant.

Also, in Japanese patients, independent predictors of their SF-6D utility scores were assessed using a repeated measures mixed linear model. The list of potential fixed-effect predictors initially included in the regression model was as follows: age (≤60 vs. ≥60), gender, obesity (defined as BMI ≥25), history of insomnia, psychiatric disorders and type 2 diabetes, hemoglobin, cirrhosis, and history of prior anti-HCV treatment. Only statistically significant fixed effect predictors were left in the model after a bidirectional stepwise selection; the significance thresholds were 0.2 for entry, 0.05 for stay.

All analyses were run in SAS 9.3 (SAS Institute, Cary, NC). Both studies (334-0118 and 337-0113) were separately approved by each site’s Institutional Review Board.

## Results

There were 153 Japanese patients enrolled in GS-US-334-0118 to receive 12 weeks of SOF + RBV (SVR 97%), and 341 patients enrolled in GS-US-337-0113 to receive 12 weeks of LDV/SOF with or without RBV (170 received LDV/SOF ± RBV with SVR = 98%, and 171 received LDV/SOF with SVR = 100%). The demographics and baseline clinical presentation of the study cohort have been reported [21]. Briefly, the cohort was 58.6 ± 9.7 years of age, 43% male, 52% treatment-naïve and 19% cirrhotic. Patients receiving different SOF-based regimens were similar in most of their characteristics except for HCV genotype and the rate of cirrhosis: those with HCV genotype 2 who were exclusively enrolled to receive SOF + RBV had a substantially lower cirrhosis prevalence (11% vs. 22%, *p* < 0.01) (Table 1).

**Table 1:** Baseline clinico-demographic parameters and health utility scores in GS-US-337-0113 and GS-US-334-0118

	LDV/SOF	LDV/SOF + RBV	SOF + RBV	*P*	All
N	171	170	153		494
Age, years	59.7 ± 9.2	59.2 ± 9.5	56.8 ± 10.1	0.0310	58.6 ± 9.7
Male	69 (40.4%)	73 (42.9%)	70 (45.8%)	0.62	212 (42.9%)
HCV genotype 1	171 (100.0%)	170 (100.0%)	0 (0.0%)	<0.0001	341 (69.0%)
HCV genotype 2	0 (0.0%)	0 (0.0%)	153 (100.0%)		153 (31.0%)
Treatment-naïve	83 (48.5%)	83 (48.8%)	90 (58.8%)	0.11	256 (51.8%)
Cirrhosis	41 (24.0%)	35 (20.6%)	17 (11.1%)	0.0097	93 (18.8%)
History of anxiety	8 (4.7%)	4 (2.4%)	3 (2.0%)	0.30	15 (3.0%)
History of depression	3 (1.8%)	7 (4.1%)	5 (3.3%)	0.44	15 (3.0%)
History of insomnia	24 (14.0%)	21 (12.4%)	21 (13.7%)	0.89	66 (13.4%)

The baseline SF-6D utility scores were identical in all three treatment regimen groups (*p* = 0.87) (Table 2). When compared between subgroups of the entire cohort, significant differences in the baseline utility scores were found between men and women (78.4 ± 11.8 vs. 74.4 ± 11.0, *p* < 0.0001) and patients with and without history of sleep disorders (71.9 ± 10.4 vs. 76.8 ± 11.5, *p* = 0.0008), but no significant difference was found between treatment-naïve and treatment-experienced patients (*p* = 0.41). Although there was a trend for those with or without cirrhosis, it did not reach statistical significance (74.4 ± 11.6 vs. 76.5 ± 11.4, *p* = 0.116). Finally, SF-D scores for patients older than 60 and younger than 60 were 74.5 ± 11.5 vs. 77.9 ± 11.2 (*p* = 0.0003).

**Table 2:** The SF-6D health utility scores (0-100) of Japanese patients with CH-C before, during and after treatment

	LDV/SOF	LDV/SOF + RBV	SOF + RBV	*p*
Baseline	76.4 ± 12.4	75.9 ± 10.3	76.1 ± 11.8	0.87
Treatment week 4	78.3 ± 12.1	75.5 ± 12.4	76.2 ± 13.0	0.10
Treatment week 8	78.8 ± 12.6*	74.4 ± 11.4	75.5 ± 13.6	0.0023
Treatment week 12	79.2 ± 12.8*	74.3 ± 12.5*	75.7 ± 13.1	0.0012
Post-treatment week 4	78.4 ± 13.1*	76.1 ± 13.3	77.4 ± 13.5	0.28
Post-treatment week 12	78.6 ± 13.0*	76.7 ± 12.3	77.1 ± 14.0	0.36

*p < 0.05 when compared to the baseline level

**Fig. 1:**
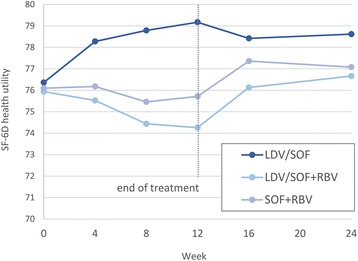
SF-6D health utility scores in Japanese CH-C patients before, during and after treatment with different treatment regimens

During treatment with RBV-containing regimens, a moderate decline in the utility scores was noted in Japanese patients receiving SOF + RBV (*p* = 0.035) (Table 2, Fig. 1). This decline, however, was completely resolved by post-treatment week 4. On the other hand, no on-treatment or post-SVR improvements in the utility scores were noted in patients treated with SOF + RBV or LDV/SOF + RBV (all *p* > 0.05).

In contrast, patients treated with RBV-free LDV/SOF experienced a rapid improvement in their average health utility scores soon after treatment initiation: *p* = 0.10 by treatment week 4, *p* < 0.05 after treatment week 8. These improvements also persisted after treatment cessation (Table 2, Fig. 1). Overall, at post-treatment week 12, a positive change from the baseline level was noted in 50.6% of all Japanese patients with HCV.

In a mixed linear model, independent predictors of lower SF-6D scores in Japanese patients included older age, female gender, and having a history of sleep disorders (Table 3). Interacted with time, receiving a RBV-containing treatment regimen was also independently associated with lower utility scores in Japanese HCV patients: beta = −5.3 for LDV/SOF + RBV, −4.1 for SOF + RBV (both *p* < 0.005).

**Table 3:** Independent predictors of health utilities in Japanese patients (*p* < 0.05 only)

Predictor	Beta	Std. Err.	*p*
Age ≥ 60 years	−4.18	0.94	<0.0001
Male gender	3.15	0.94	<0.0001
Insomnia	−6.10	1.38	<0.0001
Treatment regimen (interacted with time – end of treatment only)			
LDV/SOF	reference		
LDV/SOF + RBV	−5.33	1.32	<0.0001
SOF + RBV	−4.06	1.36	0.0030

## Discussion

This is the first study to report health utilities of Japanese HCV patients during treatment with DAAs. Notably, the most recent cost-utility studies aimed to model DAA-based treatment in Japanese HCV population relied on utility scores collected from American and European patients [25, 26]. It is reasonable to believe, however, that preference-based health utility metrics, being patient-reported outcomes per se, could be substantially affected by patients’ ethnicity, as suggested in a number of prior studies of both HCV and other clinical populations [27–31].

In this study, we showed that, at baseline, female Japanese patients with HCV, patients with history of sleep disorders, and older HCV patients all have more impairment of health utilities as determined by their SF-6D scores (Table 3). These findings are consistent with what has been already reported for other worldwide HCV populations, anti-HCV regimens, and outcomes (such as HRQL and activity scores, fatigue, work productivity) [6–9, 32, 33]. They are also important because a large number of Japanese patients with HCV are older, and older patients seem to have a moderately impaired effect of DAAs on improvement of their HRQL [34]. This may also influence cost-effectiveness of the new regimens in older Japanese patients with HCV which, however, has to be studied separately.

Our study also showed that RBV-containing regimens (SOF ± RBV or LDV/SOF ± RBV) caused a mild decrement in the SF-6D scores during treatment; however, scores soon recovered after treatment discontinuation. In contrast, the IFN- and RBV-free regimen (LDV/SOF) was found to lead to an improvement of health utilities during treatment of Japanese patients with HCV infection. In fact, in our multivariate analysis (Table 3), LDV/SOF was independently associated with higher health utility scores. Furthermore, our analysis showed that LDV/SOF is the only regimen that is associated with improvement of health utility scores post-SVR-12. This is again consistent with prior studies of utilities and other PROs in this and other populations [8, 9, 21, 33].

The study limitations include the use of the SF-6D health utility measurement which, although is widely used in cost-effectiveness studies, is still a derived score rather than a prospectively measured preference-based metric. Another limitation of our study was the small sample size of cirrhotic patients with HCV, especially those with advanced cirrhosis. Obtaining this data will be important to assess the impact of HCV infection for the entire spectrum of Japanese patients with chronic hepatitis C.

## Conclusions

In summary, we report the first health utility data for Japanese patients with HCV who were treated with the interferon-free regimens. These data can be used by health policy makers to assess the cost effectiveness of these highly effective new anti-HCV regimens in Japan.

## Abbreviations

DAA: Direct-acting antiviral
HCV: Hepatitis C virus
HRQL: Health-related quality of life
IFN: Interferon
LDV: Ledipasvir
QALY: Quality-adjusted life year
RBV: Ribavirin
SF-36: Short form-36
SOF: Sofosbuvir
